# Inertial Sensors as a Tool for Diagnosing Discopathy Lumbosacral Pathologic Gait: A Preliminary Research

**DOI:** 10.3390/diagnostics10060342

**Published:** 2020-05-26

**Authors:** Sebastian Glowinski, Karol Łosiński, Przemysław Kowiański, Monika Waśkow, Aleksandra Bryndal, Agnieszka Grochulska

**Affiliations:** Institute of Health Sciences, Pomeranian University in Slupsk, Westerplatte 64, 76200 Slupsk, Poland; karol.losinski@gmail.com (K.Ł.); kowianskip@gmail.com (P.K.); monika.waskow@gmail.com (M.W.); olka-kulczyk@wp.pl (A.B.); grochulska7@wp.pl (A.G.)

**Keywords:** inertial sensors, human gait, lumbar discopathy, computer modelling, wavelet analysis

## Abstract

Background: the goal of the study is to ascertain the influence of discopathy in the lumbosacral (L-S) segment on the gait parameters. The inertial sensors are used to determine the pathologic parameters of gait. Methods: the study involved four patients (44, 46, 42, and 38 years). First, the goal of the survey was to analyze by a noninvasive medical test magnetic resonance imaging (MRI) of each patient. Next, by using inertial sensors, the flexion-extension of joint angles of the left and right knees were calculated. The statistical analysis was performed. The wavelet transform was applied to analyze periodic information in the acceleration data. Results: in the patients with discopathy, the amount of knee flexion attained during stance phase is significantly lower than that of normal (health side), which could indicate poor eccentric control or a pain avoidance mechanism. The biggest differences are observed in the Initial Swing phase. Bending of the lower limb in the knee joint at this stage reaches maximum values during the entire gait cycle. Conclusions: It has been difficult to quantify the knee angle during gait by visual inspection. The inertial measurement unit (IMU) system can be useful in determining the level of spine damage and its degree. In patients in the first stages of the intervertebral disc disease who may undergo conservative treatment, it may also partially delay or completely exclude the decision to perform a complicated imaging examination which is MRI, often showing a false positive result in this phase of the disease.

## 1. Introduction

Intervertebral disc disease is a widespread medical and social problem [1,2]. Degeneration of intervertebral discs can lead to disc disease, commonly known as discopathy. Lumbar discopathy is the most common type of discopathy (about 95 % of all discopathy) and one of the most common complaints in adults. Practically, there are pain syndromes caused by root compression L5 and S1 [3]. The L5 root syndrome is characterized typically by the radiation of pain to the lateral surface of the thigh and an anterolateral surface of the calf to the dorsum of the foot. There is a loss of sensation on the lateral surface of the shin, dorsum of the foot, and valgus connection and the second toe. Damage to this spinal root may result in muscle weakness and/or loss of movement in the muscles of the dorsal flexors of the foot and toes, including the tibialis anterior, the short extensor of the fingers, the long extensor of the big toe, and difficulties in standing on the heels and drooping feet. Reflex elimination or weakness occurs from the posterior tibial muscle. The S1 root syndrome is characterized by pain radiating to the posterior surface of the thigh, posteriolateral surface of calf and ankle, up to the lateral edge of the foot and fifth digit. Loss of sensation may occur on the lateral edge of the foot and little finger. In this case, the triceps muscles of the calf and the plantar flexion of the fingers are affected. The gluteal muscles are also weakened. The Achilles tendon reflex is significantly weakened or suppressed [4,5].

Despite many studies, to this day medicine does not know the exact cause leading to degeneration within the intervertebral disc [6]. The essence of degenerative changes in the spine, and thus the formation of pain syndrome of the spine, is the destruction of the disc [7].

In recent years, wearable sensors, such as accelerometers and gyroscopes, have been used in the measurement of human gait analysis. These inertial sensors have the properties of lower cost, small size robustness, easiness of setting, and providing the estimation of gait parameters. Therefore, they are suitable for gait parameter estimation [8,9]. Of course, Optoelectronic Motion Capture systems (OMC) are often considered the laboratory gold standard for motion analysis of human joint kinematics due to their high measurement accuracy of 0.1 mm in the position [10]. However, most rehabilitation specialists do not even have access to a specialized laboratory equipped with such a system. OMC systems are complex to use and time-consuming in post-processing, which is why they are not implemented in everyday clinical use. Inertial Measurement Unit systems can reliably replace camera-based systems for clinical body motion and gait analyses [11]. Knee flexion is a well-known compensatory mechanism for patients with severe degenerative spine and has already been widely reported. Barrey suggested that knee flexion minimizes the importance of sagittal imbalance on full spine radiographs [12]. Measurement of knee flexion angle is mandatory. Favre et al. calculated the flexion/extension angle based on repeated alignment motions trials with an error less than 3° [13]. ProMove Mini system was tested and compared with the Motion Capture System: Tech-MCS Inertial Measurement Unit (a fully wireless motion analysis solution). Some of methods comparison IMU vs OMC were presented in [14].

We propose another, new method, based on the IMUs and wavelet analysis. Three research questions were put forward in this study.

- What are the different values of knee angle during a normal and pathological gait in four cases measured by IMU?
- Are there statistical differences of the knee’s angles in a healthy person, people with discopathy, and a person after surgical treatment?
- Could wavelet analysis (WA) be used to observe the asymmetry of a gait?

## 2. Materials and Methods

**Figure 1:**
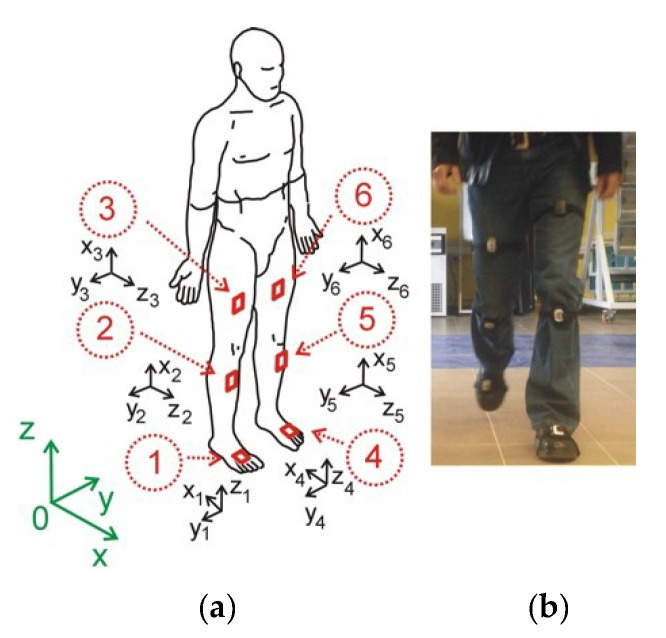
The human model and coordinate systems. The X, Y, Z coordinates represent the global coordinate system, the x_s_, y_s_, z_s_ represents the sensor coordinate system (**a**), subject wearing the ProMove mini nodes (**b**).

The experimental equipment is comprised of ProMove mini platform [15]: six ProMove mini sensors. It embeds in one device the following: 10 degrees-of-freedom inertial sensors, from 2 to 16 g accelerometer, from 250 to 2000°/s gyroscope with resolution 0.007°/s, and from 250°/s range. The flash memory of each sensor is 2 GB and low-power RF transceiver in the 2.4 GHz license-free band. Battery life is 4 h in full streaming mode. The ergonomic design of ProMove allows for easy strap attachment and body mount. Each sensor unit is 51-46-15 mm with weight 20 g (including battery). Inertia gateway as a central hub for synchronized data collection is <100 ns. The Inertia studio enables real-time visualization of sensor data, as well as over-the-air reconfiguration of the sensors and wireless parameters. All data retrieved by the Inertia Studio software is logged for post-analysis. As shown in Figure 1, the sensors are placed on the part of the body along the right and left leg on the thigh, shank, and foot. In a global coordinate system (green color), the X-axis is the walking direction, the Y-axis is the lateral direction, and the Z-axis is the opposite direction of gravity. For each sensor, the orientation is calculated relative to the Earth reference frame in terms of roll, pitch, and yaw angles. By combining the orientation of each node, we obtain the joint angles. Declaration: All procedures and measurements in this study were performed in accordance with the World Medical Association, Declaration of Helsinki—Ethical Principles for Medical Research Involving Human Subjects (version October 2013).

**Figure 2:**
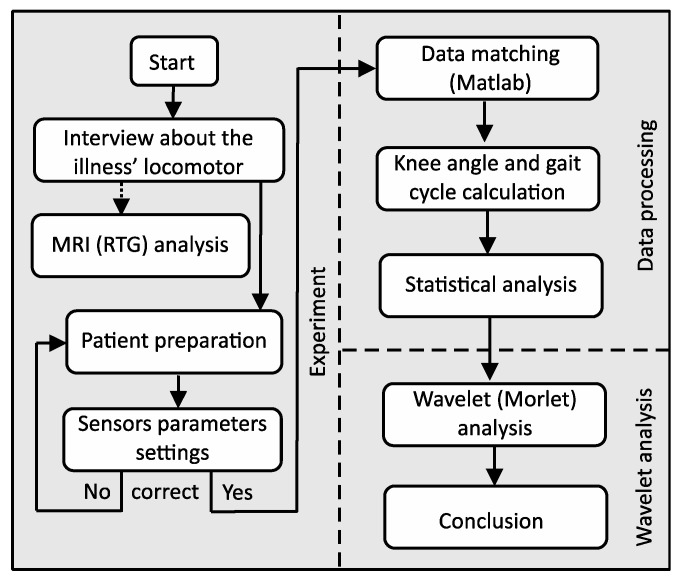
Flowchart of the experimental procedure.

Our research was divided into three parts: experiment, data processing and wavelet analysis (Figure 2). Several laboratory tests have been performed to validate the ProMove mini Inertia system (ProMove). The tests consisted in verification of walking with a Technaid wired system with implemented protocols (Motion Capture System Tech MCS) [16]. This allowed the development of wireless sensor system protocols. Prior to the start of the experiment, each person reported the state of health and past diseases that could affect the test result. Based on the data from the MRI study, we knew exactly each case report. We focus on the experiments on the following tasks:

- prepare the patient for testing (interview about the illnesses locomotor) and connect the gateway to the computer and sensor parameter settings,
- mount the sensor as shown in Figure 1, turn sensors on and verify that data is received from all of them,
- standing starting position for the examination of the patient,
- resetting the button resets the algorithm used for calculating the orientation. After resetting, it is necessary to hold the nodes still for a few seconds to stabilize their orientation;
- start recording (patient goes) and stop recording,
- collect all the raw data from all sensors to a central computer via the USB (by using the wireless system at the highest possible data rate there is packet loss). Therefore, packet loss can be avoided by enabling flash logging and downloading the logs after the experiment (via USB cable is faster),
- process the data on the computer and mark the beginning of each of the left and right step and calculation of the knee angles for the left and right leg as the arithmetic mean of several steps,
- data transformation into gait cycle and plots,
- statistical analysis,
- wavelet analysis.

In this preliminary research, each subject took 30 gait sets (10 steps in each survey). The walking speed during each set of the experiment was made to be as stable and as consistent as possible. To reduce random errors, the average step length, the stride frequency and the vertical acceleration variance for one-step were measured and calculated. The subjects were walking on a flat, even plane at a preferred walking speed. Statistical parameters were presented in Table 1. The Standardization and Terminology Committee (STC) of the International Society of Biomechanics proposed a definition of a joint coordinate system (JCS) [17]. The joint motion was determined by the orientation of each segment and was described in the XYZ-Euler angle representation. IMU’s were placed on the parallel plane to the sagittal plane. Thus, we only based on the angles in the sagittal plane. To compute the joint angle (knees), the sensor frames were transformed into a mutual coordinate system, the joint coordinate system (JCS). The rotation matrix that transforms the sensor frame at any time step into the initial sensor frame is defined by the multiplication of the single rotation matrices around each axis and is determined by

$$R_{i}=\left[\begin{array}{ccc} c\theta c\psi & -c\theta s\psi & s\theta \\ c\phi s\psi +s\phi s\theta c\psi & c\phi c\psi -s\phi s\theta s\psi & -s\phi c\theta \\ s\phi s\psi -c\phi s\theta c\psi & s\phi c\psi +c\phi s\theta s\psi & c\phi c\theta \end{array}\right]$$

where c and s denotes the cos and sin function and *ϕ*, *θ*, and *φ* are the rotation angles about U–, V–, and W– axes, respectively.

To check whether or not a model follows an approximately normal distribution, we used the Shapiro–Wilk, Lilliefors, Kołmogorov–Smirnov, and Jarque–Bera tests for normality. It is a useful and quick way of checking normality especially when we have a discrete set of data points. To apply tests to our data, we put forward two hypotheses. The null hypothesis is that sample distribution is normal. If the tests are significant, the distributions are non-normal.

The sensors transfer acceleration signals, among others. The signals consist of three components related to three directions of the Cartesian coordinate system of each sensor. The sum of the components is taken into consideration as the analyzed signal, which describes the movement of the part of a leg. The signal includes acceleration of gravity. Because the human gait is characterized by periodicities and simultaneously some characteristic periods may occur in specific periods, the wavelet tool is chosen. The advantage afforded by wavelets is the ability to perform local analysis. Because wavelets are localized in time and scale, wavelet coefficients can localize characteristic changes or differences in analyzed signals [18,19]. By shifting parameters of wavelets, they can be applied as a focus directed to the interesting signal area described by time and scale related to frequencies. The algorithm combining discrete Fourier transform (DFT) and continuous wavelet transform (CWT) has been discussed in detail in [5]. The presented procedure easily allowed to convert wavelets’ scales to frequencies, precisely to so-called pseudo-frequencies. These pseudo-frequencies represent not the exact frequencies, but some frequency ranges. The conducted analysis shows some particular features of the human gait, which are not possible to observe in raw signal (IMU acceleration signals).

## 3. Cases Description

**Figure 3:**
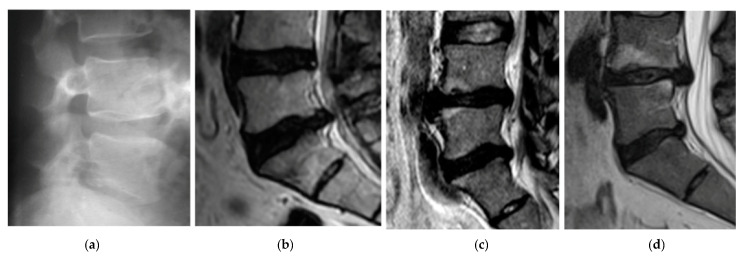
Sagittal plane magnetic resonance imaging (MRI) cases: A—case (**a**) B—case (**b**) C—case (**c**) D—case (before surgery) (**d**).

The first patient, A, was 44 years old, 1.76 m tall, and weighed 74 kg, meaning they were healthy (BMI 23.62). Diagnosis of the X-ray of the spine showed abolished physiological lumbar lordosis, a tendency to form kyphosis. The height of vertebral bodies and discs is preserved (Figure 3a). Patient A regularly engages in sports.

The second patient, B, was 46 years old, 1.77 m tall, and weighed 77 kg (BMI 24.58). The patient B has been leading a lifestyle for 10 years in which he does not play sports. He spends about 8 h a day at the computer. He complained of thigh and calf pain in the back of the limb from the 1 year. A study of MR spine, lumbosacral can be seen in Figure 3b. Other symptoms were dehydration of the intervertebral disc L5-S1, left-sided dislocation of the atherogenic nucleus to 6 mm, and the side bet filling with root pressure. Patient B participated in physiotherapy treatments and subjected to massages with without improvement. In the pharmacological treatment (in the severity of pain symptoms), patient B uses Skudexa (Tramadoli hydrochloridum 75 mg + Dexketoprofenum 25 mg) 1–2 times per month.

The third patient, C, was 42 years old, 1.75 m tall, and weighed 72 kg (BMI 23.51). He complained of paralysis of the left foot limb. A study of MR spine lumbosacral showed the smoothing lumbar lordosis and multilevel changes distorting the intervertebral joints. The symptoms of degenerative disc disease L4/L5/S1 with central protrusion disc m-k (it means “m-k”) L5/S1 entailing root compression, and the centre-left-sided disc protrusion m-k L4/L5 adjacent to the left L5 nerve root in the spinal canal were determined. The conclusion was the symptoms of lumbar disc L4/L5/S1, more the left body side at L4-L5 (Figure 3c). The disc herniation caused stretching and inflammation of an overlying nerve root. It also caused the leg pain, numbness, and tingling and weakness in the distribution of the nerve root.

The fourth patient, D, was 38 years old, 1.77 m tall, and weighed 70 kg (BMI 22.34). He complained of paralysis of the left foot limb (Figure 3d). A study of MR spine lumbosacral (2015.08.14) showed reduced intervertebral discs L4/L5, L5/S1, with reduction of their hydration, and bilateral narrowed intervertebral foramina L4/L5, L5/S1. At L4/L5 level, the central-right-side protrusion of the intervertebral disc is about 6.5 mm intrathecally with the impression of the right nerve root. At the L5/S1 level, the central-left lateral protrusion of the intervertebral disc is about 6 mm intrathecally and to the left lateral collateral. Due to severe pain and falling, foot surgery was performed on 2015/09/04. The herniation of the nucleus pulposus at the lumbar level was removed and the spinal canal was decompressed. Synthetic intervertebral baskets, struts, and threaded bone pins were introduced. Discectomy L4-L5 et stabilisatio intervertebralis posteriori columnae vertebralis eiusdem regionis BULLET TIP/2 × 9 mm × 26 mm, 1 × 10 mm × 26 mm/per fenestration protractam dextram.

In the analysed cases, B, C, and D (before the surgery), a chronic syndrome was observed (based on the interview). Patients underwent active exercises stretching the back muscles. Additional exercises consisted in strengthening the straight and oblique muscles of the abdomen, extensors of the hip joint, and flexors of the leg. Hydrotherapy and relaxation exercises in water were also used. The water environment gives almost complete relief and ensures full relaxation and stretching of the pathologically strained muscles. The patient D before surgery had a persistent impairment and compression of the spinal root and indicative of disturbances in sensation, atrophy, and paresis. All the patients have approved this research.

## 4. Results

### 4.1. Inertial Measurement Unit

**Figure 4:**
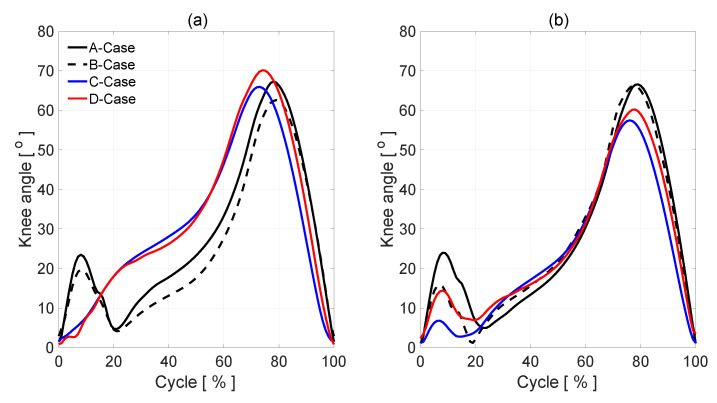
The knee angles during gait: an affected side (**a**), a healthy side (**b**).

Figure 4a,b illustrates patients’ A, B, C, and D knee angle measurements. In the normal gait, A-case, (solid line), knee flexion of the left and right leg in early stance shows a typical 2-degree flexion/extension pattern during loading, a mean knee flexion in midstance approximately 24°, a mean knee flexion in swing reached a peak of 67–68°. In B-case (dashed line, Figure 4a), after initial heel contact, the knee angle is smaller than in normal gait (the affected side). The same situation is in the healthy side. In the full extension in the healthy side, there is no difference between A and B cases. In the affected side, the knee max flexion angle is 5° smaller than in A case. In patients C and D with discopathy, the amount of knee flexion attained during stance phase is significantly lower than that of the normal (healthy side), which could indicate poor eccentric control or a pain avoidance mechanism. The knee then re-extends but not to the same amount as normal, again indicating pain avoidance or reduced control. Swing phase appears to follow a normal pattern of movement, but with a reduced amount of knee flexion at mid-swing.

In the cases of patients C and D with discopathy, the parameters defining knee bending in individual phases of gait significantly differ from the same parameters of the healthy leg. Analyzing diagram 4(a), we notice in the initial phase of the walk the lack of the deflection value characteristic for the phase of loading response. The extremity needs to perform a movement that requires very effective cooperation of the thigh muscles, in particular: tensor fascia latae, quadratus femoris, and lower legs: tibialis anterior, extensor digitorium longus, and extensor hallucis longus mostly in eccentric contraction. Lack of deflection in this phase is the result of the reduction of the strength of these muscles as well as disturbances in the integration of the proprioception system, resulting from the compression of the intervertebral disc on the nerve roots. After reaching the maximum bend, in the support phase, the knee should follow the mid-stance extension. In patients C and D, this phase does not occur. Its absence in the graph most likely indicates the patient’s avoidance of prolongation of the compressed nerve root. Then, in the terminal stance phase, we observe the greatest disproportions in patient D, whose level of infection indicates the largest losses of the most important in this phase of the muscles: gastrocnemius and soleus. Also, in the transfer phase, the results of patients C and D deviate from the norm on the part of the patient. This is manifested by a positive deviation towards the bending value, on average throughout its duration. However, the biggest differences are observed in the initial swing phase. Bending of the lower limb in the knee joint at this stage reaches maximum values during the entire gait cycle. In patients C and D, the result is in sequence 8 and 10, higher degrees than in the case of a healthy leg. In the case of patient C, this process is even more valuable, because in this case there is muscle paralysis: extensor digitorium longus, extensor hallucis longus, and tibialis posterior. These are the muscles that should keep the foot in the dorsiflexion at this stage of the gait. The lack of this bending forces knee deflection in the limb. This is another of the key phases that differentiate the level of root damage.

### 4.2. Statistical Analysis

The angle in the knee joint is considered as a continuous quantitative trait. In the beginning, a Shapiro–Wilk, Lilliefors, Kołmogorov–Smirnov, and Jarque–Bera test were performed to check if the distributions of individual curves had a normal distribution by using Statistica 13 Software [20].Therefore, a null and an alternative hypothesis were put forward.

- Null Hypothesis H0: The curves have a normal distribution.
- Alternative Hypothesis H1: The curves do not have a normal distribution.

Since at least one of the selected tests rejected the hypothesis of normal distribution, it is suggested to reject this hypothesis H0. The data do not have a normal distribution (Table 2).

Then, to estimate a rank-based measure of association, we used the Spearman’s rho statistic test. This test may be used if the data do not come from a bivariate normal distribution.

Due to the fact that the values of the $\chi_{D}^{2}$ test of each curve are in the area D of H1, there is basis for rejecting hypotheses H0. In addition, *p* < α so we have the explicitness of our decision. The optimal parameter combination was chosen to have the highest Spearman-correlation of the resulting knee angle (B, C, and D case) in comparison to patient A in affected and healthy side (Table 3). The correlation coefficients determined are significant with probability *p* < 0.05. The correlation coefficients presented in Table 2 indicate a strong positive correlation between all cases. If we pay attention to affected limbs, the highest r value is between affected C and D cases. The statistical analyses carried out indicate the positive correlation between all cases. The maximum values obtained between individual cases are marked in bold. Unfortunately, the results obtained using the Spearman’s rho statistic test do not allow for unambiguous indication of correlation features between individual cases. The reason may be too few patients tested.

### 4.3. The Gait Analysis Wavelet Concept

**Figure 5:**
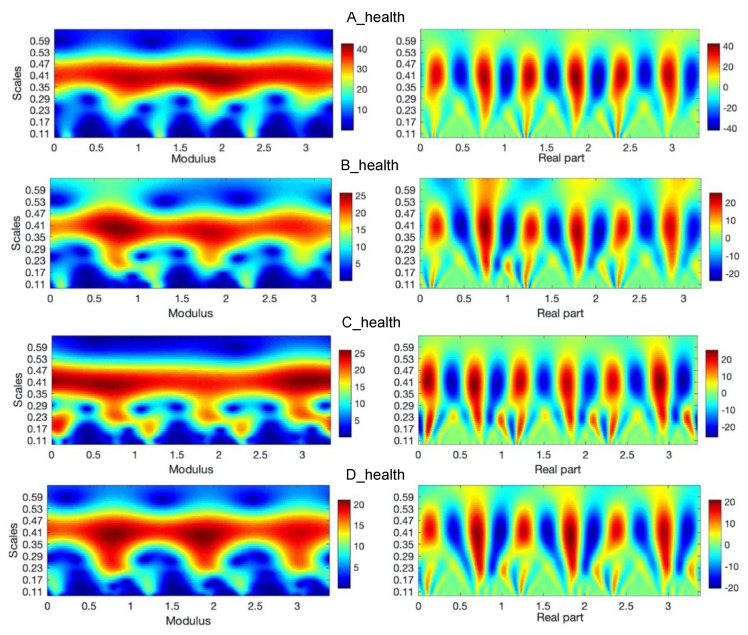
Continuous wavelet transform of the analyzed signal obtained by using Morlet wavelet of parameter 4, where modulus and real parts of wavelet coefficients are shown in case of an A-health and B, C, and D-affected leg gait.

In order to analyze gait parameters, the wavelet analysis (WA) is used in Matlab package [20]. The IMUs transfer an acceleration signal, among others. The signals consist of three components (a_X_, a_Y_, a_Z_), related to the three directions of the Cartesian coordinate system of each sensor. The sum of the components is taken into consideration as the analyzed signal, which describes the movement of the part of a leg. The signal includes acceleration of gravity. Because the human gait is characterized by periodicities and simultaneously some characteristic periods may occur in specific time periods, the wavelet tool is chosen. The advantage afforded by wavelets is the ability to perform a local analysis. The wavelets are localized in time and scale, wavelet coefficients are able to localize characteristic changes or differences in analyzed signals [18]. By shifting parameters of wavelets, they can be applied as a focus directed to the interesting signal area described by time and scale related to frequencies. A detailed methodology of this procedure in this area was presented in [5,10]. The example of wavelet analysis of three steps during a healthy gait person for sensors number 2 and 5 placed on the right and left leg is shown in Figure 5a–d. It is the analyzed signal and the signal after reconstruction by using inverse wavelet transform (WT) [16]. These analyses are conducted in the case of normal gait i.e., (nonaffected side). In the upper row of figures, one can see at first measured and next reconstructed signals (red curves), respectively for A, B, C, and D leg.

**Figure 6:**
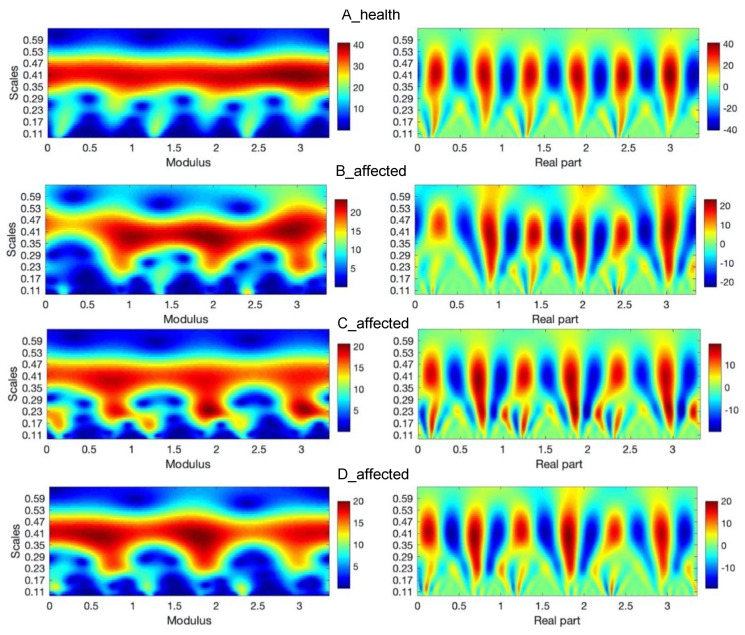
Continuous wavelet transform of the analyzed signal obtained by using Morlet wavelet of parameter 4, where modulus, real part of wavelet coefficients are shown in case of an A-health and B, C, and D-affected leg gait.

**Figure 7:**
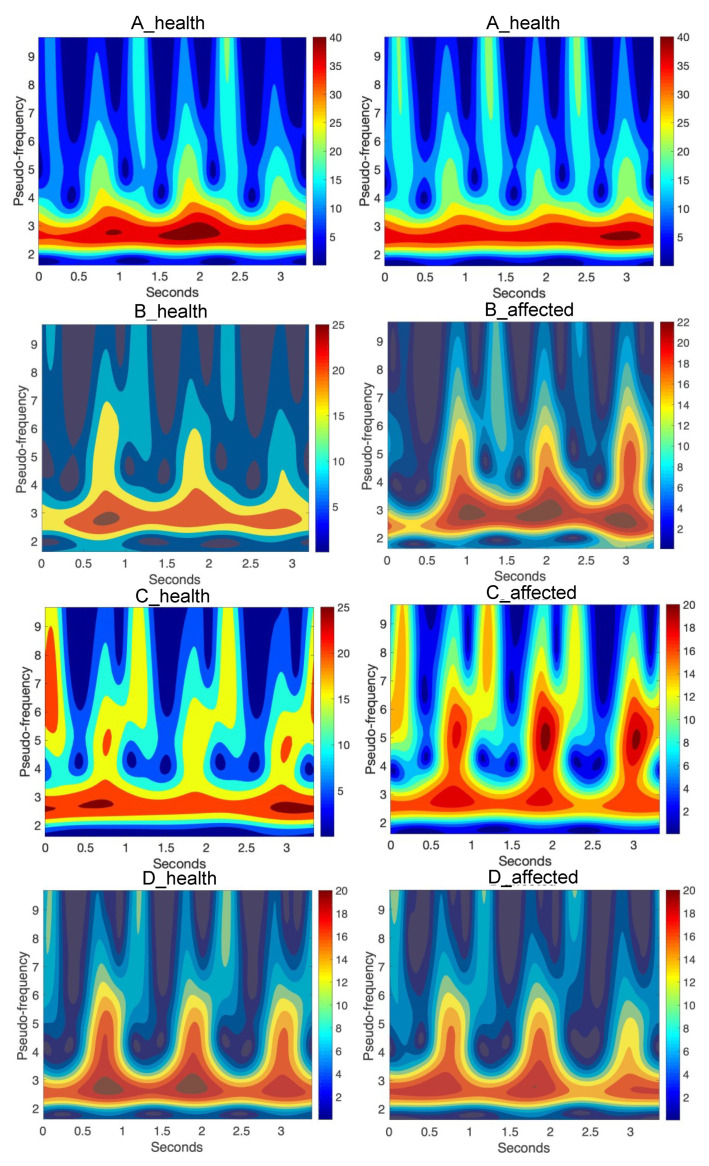
Continuous wavelet transform of the analyzed signal obtained by using Morlet wavelet of parameter 4, where pseudo-frequencies of wavelet coefficients are shown in case of A, B, C, and D patient health and affected side gait.

In the left sides of Figure 5 and Figure 6, there are absolute scales values (modulus) of wavelet coefficients shown. This kind of analysis, in a form of bands seen in these figures, may deliver details about components of a gait characterized by constant periodicity (frequencies), having variation similar to harmonic functions, for instance, sine function. In the right sides of Figure 5 and Figure 6, the real parts of wavelet coefficients are shown, which precisely show the periodicity in the 3 steps of each foot. In the side of legends of these figures, the values of coefficients change from positive maximal to negative minimal values in the clearly seen areas in these particular figures. The area approximately between 0.24 and 0.49 scales values along the whole-time axis indicate the constant pace of human gait. The character and variation of this pace confirm the harmonic components, which is clearly seen inside figures at the appropriate scales level. The other area is in the range of lower scales, at scale levels approximately below 0.24 value. The first area up to about 0.25 ms is related to steps periods, when the human foot, at first moment a heel and next to a mid-foot, take contact with the ground and next it is lifted above. Comparing the analysis in both figures (Figure 5 and Figure 6) it is possible to observe the asymmetry of a gait in A, B, C, and D cases. In the case of normal gait, (A case) the values of wavelet coefficients in the harmonic band for both legs equal 40. In the case of abnormal gait, approximately 25–20 for the left leg. It seems that some part of the energy is ”wasted” on unwanted movement. That mention above is confirmed in the case of three steps analyzed in the sequence shown in Figure 7. It is still seen from that analysis that normal gait is harmonic, which is the effect opposite to abnormal gait. In each step, the case of a person with the disease is the additional force or move component in higher frequency than the harmonic component.

This period of step is represented in the first 20% of the gait cycle, shown in Figure 4 and characterized by appropriate knee angle changes. Next, looking along the time axis at these figures, there is another region that shows a moment when body weight is moved on another leg. It allows the person to step forward. In Figure 4, it is characterized by relatively large knee angle changes. It is seen in the figures, in the case of normal gait, that the pace is nearly harmonic. The main part of the energy, which a man needs to move, is relatively evenly distributed during the step and change harmonically during this step. The symmetry of the steps is also evident in these figures. Figure 7 shows the same wavelet transform as the figure presents absolute values (modulus) of wavelet coefficients, but after recalculation to pseudo-frequencies. In Table 4, the mean of the analyzed signals as the output arguments of each case were presented.

## 5. Discussion

It has been difficult to quantify the knee angle during gait by visual inspection. However, the sensors have now made it possible to quantify the knee angle. The IMU system can be useful in determining the level of spine damage and its degree. During the course of treatment, it will monitor the progress of therapy, both in the case of improvement, centralization of the weakening of the indicator strength mm, as well as deterioration of the patient’s condition, i.e., the peripheralization of these symptoms. Objectivity of the study may find application especially for patients suffering from chronic diseases with a so-called large pain past, where the information about the location of pain is often distorted by pain memory, and also to varying degrees of body saturation with analgesics.

In the case of multi-level discopathy, which is ambiguous in the MRI image, the IMU system can be another tool in addition to measuring the indicator strength mm, reflex, paresthesia, and pain testing, which determines the level of functional damage. In patients in the first stages of the intervertebral disc disease who may undergo conservative treatment, it may also partially delay or completely exclude the decision to perform a complicated imaging examination which is MRI, often showing a false positive result in this phase of the disease.

Suggesting only this very expensive and still inaccessible imaging examination may, in this case, result in both the decision to treat the wrong level and the lack of motivation in the conservative treatment caused by the spectrum of surgery in the future. It should also be added that the latest physiotherapy methods, such as PNF, NDT Bobath, manual therapy, or McKenzie, are mainly based on functional diagnostics and can make much better use of this research primarily for the sake of its simplicity, the ability to perform almost after each therapy session, and also because it immediately shows the real progress of rehabilitation. Unfortunately, there is no such MRI, which although very accurate and irreplaceable in the case of neurosurgical interventions, after a positive end of conservative therapy expressing the complete disappearance of symptoms, often does not show significant changes in the control examination.

Shapiro–Wilk, Lilliefors, Kołmogorov–Smirnov, and Jarque–Bera tests rejected the hypothesis of normal distributions. The statistical analyses carried out indicate the positive correlation between all cases. The results are within 0.7403 (B and C affected limb) to 0.9920 (B and D healthy limb). Unfortunately, too few people were surveyed, and the results obtained using the Spearman’s rho statistic test do not allow for unambiguous indication of correlation features between individual results.

The wavelet transform was applied for the chosen signal represented by acceleration. It may be a useful tool to compare the specific details and differences in human gait. It gives information about an energy concentration during the gait and possibility to focus on the chosen part of the signal simultaneously in time and frequency domain. This feature may be a useful tool to compare the specific details and differences in human gait.

Some limitations are implied in this study. Only a small number of cases were tested. Another limit of the analysis is that it was only in a knee angle: flexion-extension (in the sagittal plane). In the current work, the cases gait was spontaneous. It would be necessary to examine healthy people and people with discopathy at various gait speeds and various stages of the disease. Long-term studies of the same people (for example, before and after surgery) would also be valuable. Obtained research results would allow creating a database related to wavelet parameters. Finally, the proposed method may be beneficial for observation of 13 symmetry of gait in discopathy patients.

## Figures and Tables

**Table 1:** Mass and kinematic gait parameters (SD—standard deviation).

Case	Height [m]	Mass [kg]	BMI	Mean Step Length [m] (SD)	Mean Stride Frequency m [Hz] (SD)	Mean Velocity (m/s) (SD)
A	1.76	74	23.62	0.83 (0.06)	1.69 (0.13)	1.40 (0.10)
B	1.77	77	24.58	0.75 (0.05)	1.43 (0.16)	1.07 (0.10)
C	1.75	72	23.51	0.73 (0.06)	1.35 (0.13)	0.98 (0.09)
D	1.77	70	22.34	0.77 (0.09)	1.58 (0.16)	1.06 (0.12)

**Table 2:** Tests of normality.

Case	Shapiro-Wilk		Lilliefors		Kołomogorov-Smirnov		Jarque-Bera	
	Stat Value	*p*	Stat Value	*p*	Stat Value	*p*	Stat Value	*p*
Ah	0.89	*p* < 0.0001	0.17	*p* < 0.01	0.17	*p* < 0.01	12.07	0.0024
Bh	0.88	*p* < 0.0001	0.16	*p* < 0.01	0.17	*p* < 0.05	11.71	0.0029
Ch	0.90	*p* < 0.0001	0.11	*p* < 0.01	0.17	*p* < 0.20	9.84	0.0073
Dh	0.87	*p* < 0.0001	0.17	*p* < 0.01	0.17	*p* < 0.01	12.34	0.0021
Ah	0.90	*p* < 0.0001	0.16	*p* < 0.01	0.16	*p* < 0.01	10.78	0.0046
Ba	0.87	*p* < 0.0001	0.19	*p* < 0.01	0.19	*p* < 0.01	13.54	0.0011
Ca	0.94	0.0003	0.08	*p* < 0.20	0.08	*p* > 0.20	5.66	0.0590
Da	0.93	0.0001	0.11	*p* < 0.01	0.11	*p* > 0.20	6.55	0.0378

**Table 3:** Spearman rank correlation coefficient between A, B, C, and D cases *p <* 0.05.

		Affected Side				Health Side			
		Ah	Ba	Ca	Da	Ah	Bh	Ch	Dh
**Affected**	**Ah**	-	**0.9920**	0.8005	0.8466	0.9813	0.9824	0.9202	0.9732
	**Ba**	**0.9920**	-	0.7403	0.7948	0.9939	0.9669	0.8800	0.9476
	**Ca**	0.8005	0.7403	-	**0.9890**	0.7167	0.8384	0.9400	0.8834
	**Da**	0.8466	0.7948	**0.9890**	-	0.7696	0.8852	0.9696	0.9245
**Health**	**Ah**	0.9813	0.9939	0.7167	0.7696	-	0.9415	0.8465	0.9204
	**Bh**	0.9824	0.9669	0.8384	0.8852	0.9415	-	0.9613	0.9920
	**Ch**	0.9202	0.8800	0.9400	0.8465	0.8465	0.9613	-	0.9810
	**Dh**	0.9732	0.9476	0.8834	0.9245	0.9204	0.9920	0.9810	-

The maximum values obtained between individual cases are marked in bold.

**Table 4:** Mean of analyzed signals.

Case	MeanSIG	Case	MeanSIG
Ah	2.95	Ah	3.10
Bh	2.43	Ba	2.23
Ch	1.81	Ca	1.30
Dh	2.30	Da	1.98
